# Measures against COVID‐19 concerning Summer Indoor Environment in Japan

**DOI:** 10.1002/2475-8876.12183

**Published:** 2020-08-22

**Authors:** Motoya Hayashi, U Yanagi, Kenichi Azuma, Naoki Kagi, Masayuki Ogata, Shoichi Morimoto, Hirofumi Hayama, Taro Mori, Koki Kikuta, Shin‐ichi Tanabe, Takashi Kurabuchi, Hiromi Yamada, Kenichi Kobayashi, Hoon Kim, Noriko Kaihara

**Affiliations:** ^1^ Faculty of Engineering Hokkaido University Sapporo Japan; ^2^ Department of Architecture Kogakuin University Tokyo Japan; ^3^ Faculty of Medicine Kindai University Osaka Japan; ^4^ School of Environment and Society Tokyo Institute of Technology Tokyo Japan; ^5^ Department of Architecture Tokyo Metropolitan University Tokyo Japan; ^6^ Shinryo Corporation R&D Center Ibaraki Japan; ^7^ Department of Architecture Waseda University Tokyo Japan; ^8^ Department of Architecture Tokyo University of Science Tokyo Japan; ^9^ National Institute of Public Health Saitama Japan

**Keywords:** ventilation, COVID‐19, infection prevention, heat stroke, coronavirus

## Abstract

Information on air‐conditioning and ventilation has been continuously disseminated in response to the Japanese Government's announcement of the need for appropriate ventilation measures against the new coronavirus disease (COVID‐19), and the issuing of an emergency presidential discourse by the presidents of Engineering Societies. In this paper, we add to the information the latest knowledge on the behavior of SARS‐CoV‐2 in air, describe its diffusion characteristics in the built environment, and summarize the effects of temperature and humidity on the virus. Then we recommend varying approaches of air‐conditioning control for facility type.

## Graphical abstract

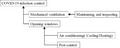

## 1 Introduction

In winter of 2019‐2020, a pandemic of COVID‐19 broke out, and the Japanese Government declared a state of emergency. As the need for cooling increases from mid‐summer to summer, it is necessary to widely disseminate appropriate ventilation methods. Therefore, the authors reviewed new papers till June 2020 on the infection of COVID‐19 in the indoor spaces, and investigated required ventilation measures which can be realized under the state of ventilation performances of Japanese buildings. Then, this paper presents reports of researchers about adequate ventilation and air‐conditioning methods, to provide measures for controlling air‐conditioners and ventilation systems in the various types of buildings for the prevention of infection and heat stroke.

## 2 Examples and Evidence of Air‐Conditioning and Ventilation Effects on Transmissibility of COVID‐19

### 2.1 Cases of infection in which environmental factors seem to have promoted infection

#### 2.1.1 Restaurant in Guangzhou, China

**Figure 1:**
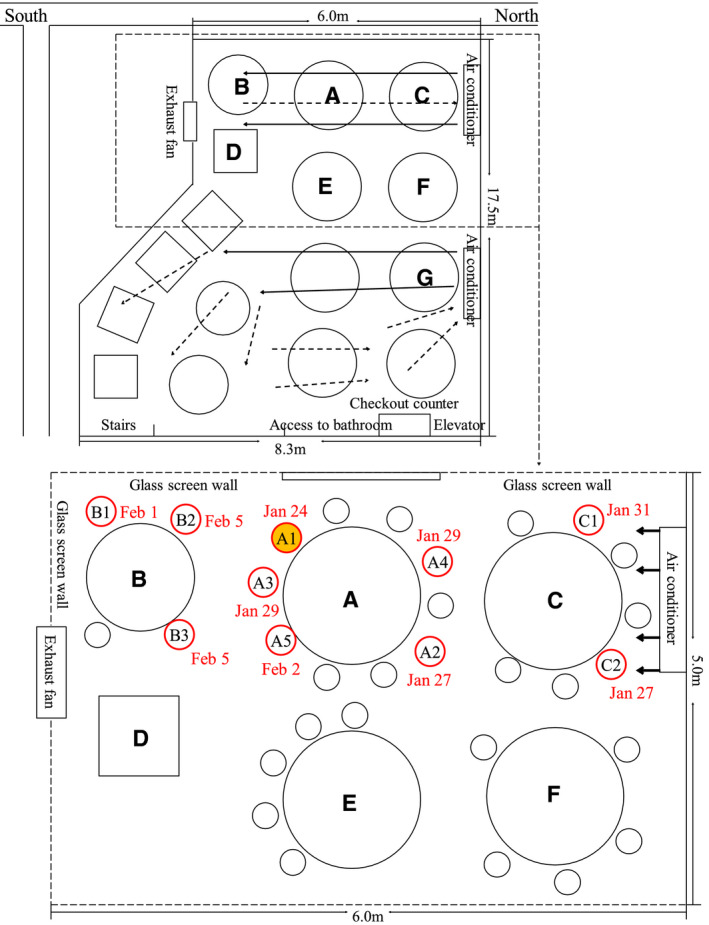
Sketch showing arrangement of restaurant tables of COVID‐19 outbreak, Guangzhou, China, 2020. Red circles indicate seating of future case‐patients; yellow‐filled red circle indicates index case‐patient. Adapted from Lu 2020^1^

On January 24, 2020, a novel coronavirus infection occurred in a restaurant in Guangzhou, China, infecting 10 people in three families.^1^ The infected person (A1) had lunch with 9 friends for about an hour to an hour and a half. Fifty percent (5 out of 10) of those at the same table as the infected person were found to be infected within the next 7 days. In the adjacent leeward table, 75% (3 out of 4) were infected. Two of the seven people on the windward table were infected. There was no infection from the air‐conditioner to the exhaust fan outside the main airflow (Figure 1).

The authors concluded that air currents in the air‐conditioners promoted droplet infection, and recommended that distances between people are maintained and ventilation improved.

#### 2.1.2 Call center in South Korea

**Figure 2:**
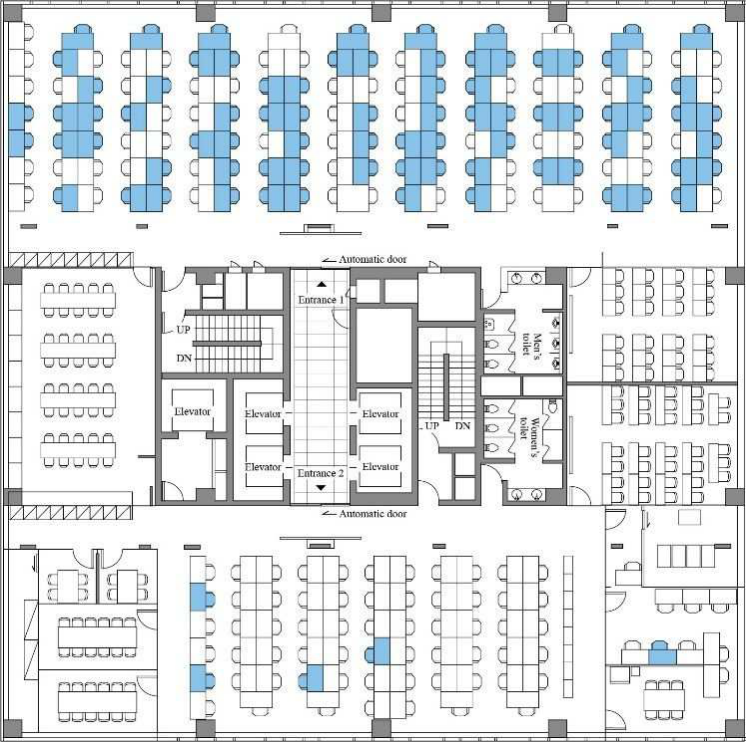
Floor plan of the 11th floor of the building, site of a coronavirus disease outbreak, Seoul, South Korea, 2020. Blue coloring indicates the seating places of persons with confirmed cases. Adapted from Park 2020^2^

One infected person went to work on the 11th floor of a mixed commercial and residential building.^2^ The floor has 216 employees, 94 of whom (43.5%: indicated by blue Seats) were infected during the 2‐week period between February 25, 2020—when an infected person working on the 11th floor developed symptoms—and March 9, 2020—when the building closed. Of the 94 patients, 92 developed the disease and only 2 were asymptomatic. The fact that one side of the office was predominantly infected, while the other had very few infected individuals, suggests that SARS‐CoV‐2 is very likely to have spread in a crowded office environment such as a call center. It also points out that although there may have been many contacts with workers on different floors in elevators, lobbies, etc., the infection was mostly confined to one floor, and that contact time may be a major factor promoting the spread of SARS‐CoV‐2 (Figure 2).

#### 2.1.3 Washington State Squadron practice

Of the 61 people who participated in choir practice on March 10, 2020, 53 cases were identified, including 33 confirmed cases and 20 possible cases, among those experiencing one symptom of COVID‐19.^3^ The secondary infection rate was 53.3% from confirmed cases and 86.7% from all cases. Three of the 53 patients were hospitalized (5.7%) and 2 died (3.7%). During the 2.5‐hour singing practice, members sat close to each other, and at the end of the practice, they piled up chairs, thus increasing the chance to be infected by aerosols or by contact. The act of singing itself may have contributed to the infection by the release of aerosols, related to the loudness of the voice.

#### 2.1.4 Features common to cases of cluster infection in Japan

Houseboat, live houses, exhibition halls, sports gyms, medical facilities, welfare facilities, restaurants, etc., are considered to be closed, poorly ventilated, and crowded environments.^4^, ^5^ However, it is necessary to investigate and examine how ventilation was carried out in the event of an outbreak.

### 2.2 Environmental survey

#### 2.2.1 Diamond Princess cruise ship

In February 2020, an outbreak of COVID‐19 occurred on the Diamond Princess cruise ship. A total of 712 patients were confirmed among the 3711 passengers and crew members it was carrying. The National Institute of Infectious Diseases of Japan conducted an in‐ship environmental survey to determine the degree of environmental contamination around the patient and to estimate the transmission of infection. Swabs were used to wipe the environmental surfaces of 97 common areas and 490 locations in 49 passenger and crew rooms. SARS‐CoV‐2 ribonucleic acid (RNA) was detected in samples by reverse transcription polymerase chain reaction (RT‐PCR). Air samples were also collected from 14 sites in 7 rooms and subjected to the same tests. The maximum period of time between passengers and crew leaving the room and sampling from the room was 17 days. SARS‐CoV‐2 RNA was often detected from environmental surfaces around the toilet, desk, telephone, TV remote controller; and as a result, it was reported that the environment around infected persons was contaminated, regardless of the presence or absence of symptoms of SARS‐CoV‐2. It was not detected from the rooms of persons who were not infected, nor in air samples. Although there was no evidence to suggest airborne transmission, SARS‐CoV‐2 RNA was detected in the hallway ceiling exhaust, and further investigation is needed on the possibility of the virus floating away in special environments.^6^ The U.S. Centers for Disease Control and Prevention (CDC) reported that as of February 8, 2020, there was no evidence of room‐to‐room transmission of viruses via the air handling unit on board the Diamond Princess cruise ship.^7^ The primary route of transmission for the outbreak was by droplets and contact. There was no evidence of long‐distance airborne transmission through central air conditioning systems during the outbreak.^8^

#### 2.2.2 Huoshenshan Hospital in Wuhan, China

Surface and air samples were collected at two Wuhan hospitals and quantitative real‐time PCR tests were performed. SARS‐CoV‐2 RNA was found to be widely distributed on the surface of floors, computer mouses, garbage receptacles, and sickbed handrails. It was also detected in 35% (14/40) of air samples taken from ICUs and in 12.5% (2/16) of samples from up to 4 m away from the patient in general wards. In addition, it was reported that the detection rate was high near the patient or the exhaust port, and that the amount of virus detected decreases when the personnel density in the space decreases.^9^

#### 2.2.3 The Renmin Hospital of Wuhan University, Wuchang Fanggang Field Hospital, Wuhan, China

Airborne SARS‐CoV‐2 RNA levels detected in isolated and ventilated rooms were very low, but were elevated in the patients' toilet areas. High levels of viral RNA were detected in some medical staff areas, but dropped to undetectable levels after strict eradication procedures were performed. Although the infectivity of the detected virus was not confirmed, it was suggested that SARS‐CoV‐2 may be transmitted through aerosols. In most of the public areas, airborne SARS‐CoV‐2 RNA was detected in two more congested areas.^10^

#### 2.2.4 Singapore SARS‐CoV‐2 Outbreak Dedicated Center Toilet

The adhesion of viruses, which are considered to be present as a result of contact, was detected in toilets.^11^ It has been suggested that toilet droplets may have been the source of infection for SARS,^12^ and there have been reports of 23% of patients with negative airway results testing positive for SARS‐CoV‐2 in their stools.^13^ Attention therefore should also be paid to transmission by toilet droplets.

#### 2.2.5 The University of Nebraska Medical Center

During initial isolation of 13 patients identified as COVID‐19 positive at the University of Nebraska Hospital, air and surface samples were collected in 11 isolation rooms to investigate viral spread from isolated patients. Evidence of viral contamination in many commonly used articles, toilet facilities, isolated cubicles, and corridor air samples showed that SARS‐CoV‐2 was introduced into the indoor environment via exhaled air particles, stool, and contact with environmental surfaces. The RNA concentration of SARS‐CoV‐2 in the air near the window away from hospital room patients, was 3.76 copies/L.^14^

#### 2.2.6 Cases where SARS‐CoV‐2 was not detected in air samples

Faridi et al. reported that testing samples taken at a distance of 2‐5 m from the patients' beds in 10 rooms at an Iranian hospital did not detect SARS‐CoV‐2. However, the amount of air sampled was as low as 90 L.^15^ Also, Ong et al. found no SARS‐CoV‐2 activity in hospital room air samples but found activity in samples taken from the exhaust surfaces in a Singapore hospital infection control room.^11^ A similar study found SARS‐CoV‐2 RNA in environmental surface samples but not in air samples in airborne infection isolation rooms with 12 air changes/hour (ACH).^16^ Considering that indoor air is discharged by the ventilation system, these results suggest that SARS‐CoV‐2 should be present viable in the air. The isolation room examined by Faridi et al. and Cheng et al. had a ventilation rate of 12 ACH, suggesting that even when present in air, it was diluted by ventilation and fell below the detection limit.

These results indicate that adequate ventilation contributes to the reduction of airborne concentrations of SARS‐CoV‐2. In addition, since the presence or absence of infection cannot be determined only from the results of environmental surveys, further research on the routes of infection in each environment is necessary.

### 2.3 Environmental conditions and survival rate of SARS‐CoV‐2

The stability of SARS‐CoV‐2 was comparable to that of SARS‐CoV in the experimental environment. As an aerosol with a particle size of less than 5 μm, its survival time was observed to be about 3 hours (Halving time: about 1 hour), and in its state of adhesion to surfaces, its survival time was determined at about 1‐3 or 4 days, depending on the material it adhered to.^17^

The effect of temperature and humidity on SARS‐CoV‐2 in the general environment is still largely unknown, but in experiments with Coronavirus Transmissible Gastroenteritis Virus, the inactivation of surface‐attached viruses 20°C increased is steeply at 20°C compared with those at 4°C, regardless of humidity. For relative humidity, survival rates were highest at 20% and 80%, and lowest at 50%.^18^

### 2.4 Relationship between environmental conditions and COVID‐19 infection

Regarding COVID‐19, negative or positive correlations with air temperature, and negative correlations with humidity (high humidity reduces risk) have been reported.^19^, ^20^, ^21^, ^22^, ^23^ Thus, the same tendencies as seasonal influenza have been observed.

### 2.5 Pathways of infection

#### 2.5.1 WHO reports

The authors note that the main modes of transmission of COVID‐19 virus are infection through droplets and contact, and that airborne transmission can occur in environments where there are higher levels of aerosols generated. Regarding the detection of SARS‐CoV‐2, they noted that some media reports have suggested airborne transmission, and pointed out that it is important to know whether an active virus has been detected and what role it plays in the transmission, and that the information should be interpreted carefully.^24^

Epidemiological evaluations of more than 75 000 COVID‐19 patients in China have similarly reported the route of infection. Reports of COVID‐19 infection in prisons and long‐term care facilities in closed environments indicate that close proximity of people, contact with people in these environments, and the potential for environmental pollution are important factors that can amplify the infection, and call for further research on these aspects.^25^

In closed spaces where many people gather, there is a possibility of airborne infection, and it is necessary to consider these situations in the future.

#### 2.5.2 Importance of ventilation for infection control

Morawska et al. warned of the spread of infection via air and pointed out the importance of ventilation in situations where the need for ventilation as an infection control measure is not recognized by countries or authorities.^26^

#### 2.5.3 Analysis of air flow at Guangzhou restaurant in China

The spread of infection in restaurants has been reported to be caused by droplet transmission, but it is also proposed that droplet transmission alone cannot explain the outbreak. If the 10 reported infections occurred at the distances shown in Figure 1, airborne transmission should also be suspected. Airborne droplets dry out within a very short period of time, so it is possible that the droplet nuclei have caused the infection. Yuguo Li et al. obtained a video record of the lunch, and conducted ventilation measurements and numerical analysis to examine the outbreak. They found there were no exhaust fans running during the lunch and the measured ventilation rate was 0.75‐1.04 L/s per person. They pointed out that the infection could not be explained only by the airflow, and the ventilation failure contributed to the high infection rate. They concluded that aerosol transmission of SARS‐CoV‐2 due to poor ventilation may explain the spread of COVID‐19.^27^

#### 2.5.4 Items common to influenza pandemic periods

In Okinawa, seasonal flu outbreaks peak twice—in winter and summer.^28^, ^29^, ^30^, ^31^ An increase in the number of people and time spent in air‐conditioned rooms with windows closed is considered to be a common feature during the epidemic season. Lack of ventilation in rooms with inadequate mechanical ventilation may increase the exposure of infected persons to droplets and droplet nuclei, and promote the spread of infection.

It can be seen from the above that the SARS‐CoV‐2 diffuses far into the indoor air, but the concentrations are low when sufficient ventilation is provided. The main routes of transmission of COVID‐19 due to SARS‐CoV‐2 are contact infection and droplet infection, and there have been no reports of airborne infection so far.^32^ This may be because the concentration of SARS‐CoV‐2 had decreased during its spread and had not reached a level sufficient to cause disease. WHO also points out the importance of reducing infectious virus concentrations in indoor air.^33^ However, in poorly ventilated spaces, concentrations of the virus in indoor air may be high, and may pose a risk of infection. Although this situation was previously thought to occur only in hospitals where aerosol‐generating procedures such as sputum induction were performed, cases of COVID‐19 outbreaks analyzed indicate that similar risks may occur in areas with three conditions; closed spaces with poor ventilation, crowded places, and close‐contact settings such as those where close‐range conversations overlap.^34^ It has also been estimated that patients subjected to these three conditions are 18.7 times more susceptible to infection.^4^ Therefore, countermeasures by ventilation are necessary.

### 2.6 Minimum ventilation rate

Elucidation of the transmission mode of SARS‐CoV‐2 requires considerable time and expense. It is important to take preventative measures against possible risks of infection in the first place. In particular, cases have been reported in which droplet infection is suspected due to the promotion of droplet infection or droplet nuclei in poorly ventilated spaces or in environments where many people gather.

With regard to SARS‐CoV‐2, since there are limited data available, and it is difficult to set the minimum required ventilation rate based on the evidence obtained thus far. However, if appropriate ventilation is provided to maintain the standards stipulated in the Act on Maintenance of Sanitation in Buildings,^35^ such as a CO_2_ concentration of 1000 ppm or less, the risk of airborne infection is not considered to be high, on the assumption that personnel density is controlled and distance between people is ensured. In addition, it is desirable to increase the ventilation rate (Outside air intake) to an acceptable range from the viewpoint of the thermal comfort of occupants.

### 2.7 The role of HVAC system in infectious aerosols control

#### 2.7.1 Possibility for controlling infectious aerosols by ventilation

WHO defines droplets as respiratory aerosols >5 μm in diameter; and droplet nuclei as the residue of dried respiratory aerosols, which are ≤5 μm in diameter and result from evaporation of droplets coughed or sneezed into the atmosphere or by aerosolization of infective material.^33^ Based on the field measurement results of Wuhan Hospitals during the COVID‐19 outbreak, Liu reported that the peak concentration of SARS‐CoV‐2 aerosols appears in two distinct size ranges—one in the submicron region with aerodynamic diameters dominant between 0.25 and 1.0 µm, and the other in the supermicron region with diameters larger than 2.5 µm.^10^ The main generation sources of SARS‐CoV‐2 are coughing and sneezing by patients. Saliva droplets can travel long and far in airflow, depending on their sizes. Most communicable respiratory infections are transmitted via large droplets within short distance or by contact with contaminated surfaces. Large droplets (diameter >60 μm) tend to settle quickly from the air, so the risk of pathogen transmission is limited to individuals in close proximity to the saliva droplet source. Small droplets (diameter ≤60 μm) may cause short‐range transmission (when the distance between individuals is less than 1 m). Small droplets are likely to evaporate into droplet nuclei (diameter ≤10 μm) in favorable environments, thus increasing their potential for long‐distance aerosol transmission.^36^

The suspension of particles in air is an important factor in infectiousness of aerosols. Terminal settling velocity, a condition where the drag force of the air on the particle will be exactly equal and opposite to the force of gravity,^37^ is proportional to the square of particle diameter, and therefore increases rapidly with particle size. Equation (1) for terminal settling velocity *v* is given as:

(1) $$v=\frac{gD_{p}^{2}\rho_{p}}{18\mu }$$

where *g* [cm/s^2^] is the acceleration of gravity, *D_p_* [cm] is the particle diameter, *ρ_p_* [g/cm^3^] is the density of the particle, and *η* [g/(cm·s)] is the viscosity.

**Figure 3:**
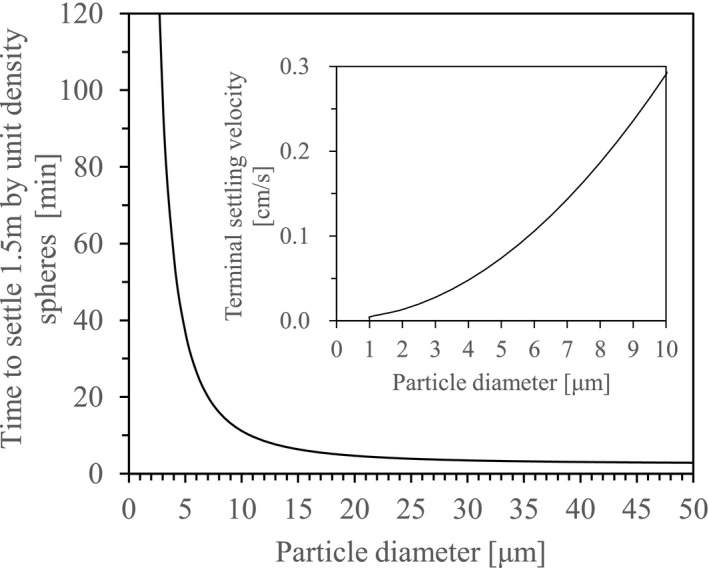
Terminal settling velocity and the time to settle 1.5 m in still air

Most droplets released from people's mouths contain moisture. Thus, assuming that a particle density is 1 g/cm^3^, the terminal settling velocity and the time to settle 1.5 m in still air can be calculated for different particle diameters (Figure 3). It turns out that particles ≤10 μm are suspended in still air for a longer time (1 μm for 14.4 hours; 5 μm for 35 minutes; and 10 μm for 9 minutes). Moreover, terminal settling velocity of particles ≤10 μm is lower than 0.3 cm/s (0.003 m/s), and is also based on indoor relative humidity. Since particle diameters become smaller by evaporation of the droplets during settling, it will be suspended indoors for a longer time. Therefore, aerosols ≤10 μm are easily transported over a long‐range (even up to the inlet air) in the indoor airflow during the operation of air‐conditioning/ventilation equipment. Moreover, the standard value of air velocity according to the Act on Maintenance of Sanitation in Buildings (MHLW, Ministry of Health, Labour and Welfare, 1970) is ≤0.5 m/s, and field measurement results show the highest and average velocities in occupant spaces are 0.4 m/s and 0.1 m/s, respectively.^38^, ^39^ This implies that it is possible to control the aerosols containing viruses by a proper indoor airflow plan.

**Figure 4:**
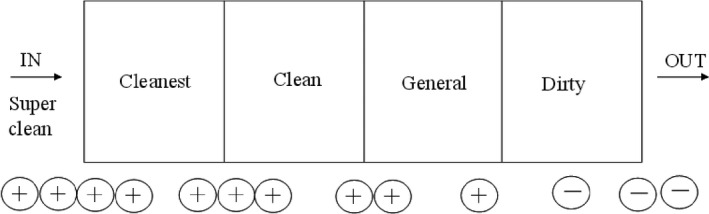
Controlling air movement via pressure relationships

#### 2.7.2 Ventilation strategies

As larger droplets generated by coughs and sneezes generally affect other persons only within 1‐2 m, it is virtually impossible for HVAC (Heating, Ventilation, and Air‐Conditioning) system to exert control for close exposure. If an appropriate design and management is carried out, HVAC can control the small droplets or droplet nuclei. Considering the dissemination range of coughs and sneezes, particle diameter characteristics of SARS‐CoV‐2, sedimentation velocity of aerosols, and indoor airflow, it can be said that ventilation is the most effective method for reducing the concentration of SARS‐CoV‐2 contained in aerosols ≤10 μm. Furthermore, some previous studies show that droplets from human respiration activities are mostly <5‐10 μm in diameter.^40^, ^41^, ^42^, ^43^, ^44^, ^45^, ^46^, ^47^, ^48^, ^49^

If it assumed that the generated contaminant is diffused uniformly indoors and the indoor air is in a perfect mixing state, then steady state concentration *C* gives as:

(2) $$C=Co+\frac{M}{Q}$$

*C*, Indoor pollutant concentration [mg/m^3^] or [/m^3^]; *Co*, Initial indoor pollutant concentration [mg/m^3^] or [/m^3^]; *M*, Indoor pollutant generation [mg/h] or [/h]; *Q*, Air change rate [m^3^/h].

Since indoor concentration is inversely proportion to air change rates (Equation 2), indoor concentration will decrease with increasing air change rates. In real conditions, the indoor environment is not in the ideal state mentioned above. Therefore, to raise the efficiency of ventilation, a proper airflow plan becomes important.

Mechanical ventilation systems are capable of providing controlled ventilation to occupant space. On the other hand, specialized natural ventilation systems may be applicable to a wider range of climatic conditions and buildings including large commercial buildings. Much depends on individual circumstances and requirements. For a given configuration of openings, the natural ventilation rates vary according to the prevailing driving forces of wind and indoor/outdoor temperature difference.^50^ Since the airflow plan of natural ventilation is difficult to predict, it needs to be a cautious cognizant of adverse currents of infectious aerosols.

#### 2.7.3 Filtration

The use of highly efficient particle filtration in centralized HVAC systems reduces the airborne load of infectious particles.^51^, ^52^ An air filter collects suspended particles near the filter media by mechanisms such as inertial collision, interception, diffusion, and electrostatic attraction. In fact, particle collecting by an air filter is based on one or more of these mechanisms. The mechanisms differ depending on the particle size. The collecting efficiency increases with inertial impaction of large particles and with diffusion of small particles. It is lowest for particles with an approximate size of 0.2‐0.3 μm. The collection efficiency of air filters for suspended particles by particle size is shown in the Table 1. In office buildings, medium efficiency air filters (equivalent to MERV11‐13 in Table 1) are typically used, and a high quality (i.e., MERV12) is sufficient to remove >90% of the large droplet nuclei.^53^ High efficiency particulate air filters (HEPA) with 99.97% or higher particle collection efficiency for particles with the size of 0.3 μm at the rated airflow volume are used for rooms such as hospital operating rooms that demand high air cleanliness (JIS Z 8122).

**Table 1:** Minimum efficiency reporting values (MERVs) and filter efficiencies by particle size

MERV	0.3‐1.0 µm	1.0‐3.0 µm	3.0‐10 µm
1	n/a	n/a	E_3_ < 20
2	n/a	n/a	E_3_ < 20
3	n/a	n/a	E_3_ < 20
4	n/a	n/a	E_3_ < 20
5	n/a	n/a	20 ≦ E_3_
6	n/a	n/a	35 ≦ E_3_
7	n/a	n/a	50 ≦ E_3_
8	n/a	20 ≦ E_2_	70 ≦ E_3_
9	n/a	35 ≦ E_2_	75 ≦ E_3_
10	n/a	50 ≦ E_2_	80 ≦ E_3_
11	20 ≦ E_1_	65 ≦ E_2_	85 ≦ E_3_
12	35 ≦ E_1_	80 ≦ E_2_	90 ≦ E_3_
13	50 ≦ E_1_	85 ≦ E_2_	90 ≦ E_3_
14	75 ≦ E_1_	90 ≦ E_2_	95 ≦ E_3_
15	85 ≦ E_1_	90 ≦ E_2_	95 ≦ E_3_
16	95 ≦ E_1_	95 ≦ E_2_	95 ≦ E_3_

n/a: not available.

The maintenance of the air filter can be performed as usual for full outside air operation. For return air operation, it is recommended to check the differential pressure of the filter often and replace the filter sooner than usual, so that the dust collected by the filter will not pass through and enter the room.^54^

#### 2.7.4 Effective use of an air purifier

There are two types of air purifiers targeting suspended particles—a filtration type, and an electric dust collection type in which the particles in the air are charged when passing through the ionization section and then collected by the electrostatic precipitator located behind it. The latter is mainly for commercial use. In recent years, an ion‐releasing type air purifier has also been created; however, it is reported that its effectiveness in reducing active virus in the air was much lower than any existing filtration technologies.^55^ On March 10, 2020, the Consumer Affairs Agency urgently requested businesses to improve their labeling with respect to their claims about negative ion generators and ion air purifiers providing protection against COVID‐19.^56^

This article focuses on filtration‐type air purifiers. The mechanism of filtration in a filtration‐type air purifier is the same as in the air filter described above. In the case of an air filter installed in an air‐conditioner, almost all air is supplied to the indoors through the air filter with little leakage. In contrast, an air purifier filters suspended particles in the air, while circulating the indoor air. Therefore, the purification performance of a filtration‐type air purifier depends on not only the filter's collection efficiency but also its airflow volume. The Equation (3) shows the composition of indoor pollutants along with their concentrations in the case of installation of a filtration‐type air purifier. The purification performance of an air purifier is determined by *qη/V*. Accordingly, it is necessary to determine the number of air purifiers and airflow volume considering the room volume. The use of an air purifier is effective as auxiliary equipment. However, when the ventilation volume can be secured, it can provide greater reduction of the virus concentration.^54^

The Equation (3) targeted the infectious aerosols ≤10 μm which transport over a long range. Moreover, the Equation (3) is premised on contaminants generated continuously. As mentioned above, the main generation sources of SARS‐CoV‐2 are coughing and sneezing by infected people. Therefore, since the generationg of SARS‐CoV‐2 is intermittent, the concentration *C* becomes lower than the case of hypothetic continuous generating. WHO described transmission of SARS‐CoV‐2 as follows: current evidence suggests that transmission of SARS‐CoV‐2 occurs primarily between people through direct, indirect, or close contact with infected people through infected secretions such as saliva and respiratory secretions, or through their respiratory droplets, which are expelled when an infected person coughs, sneezes, talks or sings. Some outbreak reports related to indoor crowded spaces have suggested the possibility of aerosol transmission, combined with droplet transmission, for example, during choir practice, in restaurants or in fitness classes.^57^

(3) $$C=Coe^{‐\frac{q\eta }{V}t}+\frac{M}{q\eta }\left[1‐e^{‐\frac{q\eta }{V}t}\right]$$

*M*, Indoor pollutant generation [mg/h] or [/h]; *V*, Room volume [m^3^]; *C*, Indoor pollutant concentration [mg/m^3^] or [/m^3^]; *Co*, Initial indoor pollutant concentration [mg/m^3^] or [/m^3^]; *η*, Air purifier collection efficiency [‐]; *t*, Elapsed time [h]; *q*, Air purifier airflow volume [m^3^/h].

#### 2.7.5 Temperature and relative humidity

To date, much research has reported on the influence of relative humidity (RH) on coronaviruses. Shechmeister and Shaffer found that survival of airborne influenza virus was maximal at 20%‐25% RH, minimal at 50% RH, and moderate at 70%‐80% RH.^58^, ^59^ Mousavi reported that scientific literature generally reflects the most unfavorable survival for microorganisms when the RH is between 40% and 60%.^51^

The impacts of air temperature and RH on SARS‐CoV‐2 in indoor environment is not well understood as yet. Casanova reported that inactivation of adhesion coronaviruses (such as transmissible gastroenteritis virus and mouse hepatitis virus) was more rapid at 20°C than at 4°C, at all humidity levels.^18^ The relationship between inactivation and RH was not monotonic, as there was greater survival at low RH (20%) and high RH (80%), than at moderate RH (50%). Taking into consideration the above‐mentioned findings on airborne and adhesion virus experiments, it is estimated that the survival of coronaviruses is the lowest under the conditions of an RH of about 50%. On the other hand, regarding temperature, high temperatures reduce the probability of survival of a virus. Considering the actual conditions in indoor environments, it would be more realistic to control RH than temperature for creating conditions to destroy the virus.

## 3 Varying Approaches of Air‐Conditioning/ Ventilation Control for Facility Type

### 3.1 Office buildings

Centralized air‐conditioning systems, as a principle, should be adjusted to increase the intake of air volume. Additionally, the volume damper openings of the outside air supply fan and exhaust fan should be increased while paying attention to the air balance. In cases where the air flow is inverter‐controlled, one should raise the current value and inverter of the supply and exhaust fans and remove the automatic control of the external air volume, keeping the constant air volume damper of the external air system open. If the fan is motor‐driven through the pulley, one must exchange it with a large diameter pulley on the motor side. The ventilation volume should be increased by selecting the strong operation mode as long as the noise generated is acceptable. Updating the filter of the outside air system may also increase the air flow volume. Operation should be as close to full outside air operation as possible, to minimize returned air volume from indoors and prevent the virus from re‐entering the room through the returned air.

In ventilation equipment of buildings with CO_2_‐concentration control, lowering the indoor CO_2_ concentration setting (normally at 1000 ppm) can increase the ventilation volume (it will be maximized when the setting is lower than the outside concentration). If there is a mode for outside air cooling, it should be adjusted to preferentially operate outside air cooling by raising the upper limit of the outside air cooling permission conditions and lowering the lower limit. If there is a timer to control this operation, it should be adjusted to extend the operation time to longer than the occupancy time in the room. Operations should be started several hours earlier than usual, and turning off should be delayed in case of any remaining occupants, and if possible, operated continuously for 24 hours.

If there is a total heat exchanger, similar measures can be taken for the stationary type as for the individual ventilation system. For the rotary type, if a purge sector is set and the pressure balance is adjusted properly, that is, return air pressure < supply air pressure, then the risk of virus entry is considered to be low. Therefore, it is recommended to operate in a mode to allow a large effective ventilation volume, while checking/adjusting the operation status, as necessary.^54^

On the other hand, with individual air‐conditioning systems, introduction of the outdoor air by a fan is often separated from a package type air‐conditioner. In this case, it is important to operate it without forgetting about the outdoor air fan. Moreover, since the filtration efficiency of the filter fitted in a package type air‐conditioner is generally low, the use of a portable air purifier will be effective as an auxiliary device.

Regarding temperature and RH, the principle is to satisfy the standard value (temperature: 17‐28°C; RH: 40%‐70%) recommended in the Act on Maintenance of Sanitation in Buildings. Moreover, if possible, RH should be controlled in 40%‐60%.

### 3.2 School

A COVID‐19 cluster generated in a school has been reported in Japan. Since the density of students in a classroom is high, social distancing is most important. In Japan, a school with a total floor area ≥8000 m^2^ has specific building requirements in terms of the Act on Maintenance of Sanitation in Buildings. Therefore, it is necessary to satisfy the related standards according to the law, such as temperature, RH, and CO_2_ concentration. In addition, the indoor environment should be controlled by referring those standards. The school also needs to control ventilation, temperature, and RH based on the “School environmental hygiene management manual, revised edition in Heisei 30 fiscal year” released by Ministry of Education, Culture, Sports, Science and Technology.^60^

With respect to mechanical ventilation, as mentioned for office buildings, the principle is to increase air change rates. On the other hand, the ventilation efficiency will improve if windows on the outside of a classroom, and windows (in certain cases) facing the passage, are opened on days when natural ventilation is possible. On days when natural ventilation is impossible, a ventilator should be operated to introduce sufficient outdoor air.

Regarding the control of temperature and RH, the principle is to satisfy the standard value of the Law for Environmental Health in Buildings. Moreover, if possible, RH should be maintained in the range 40%‐60%.

### 3.3 Museum

The exhibition room of a museum is a space the demands on temperature and RH, along with high air cleanliness factor, and is accordingly fitted with HVAC equipment. Introduction of outdoor air to the exhibition room and management of an air filter should be carried out similar to that in the office building mentioned above. The temperature and RH should be set according to not only occupants' health and comfort, but also to the exhibits. Therefore, management should balance both the needs of creating a suitable indoor environment for exhibits and for accommodating 40%‐60% occupants.

### 3.4 Theater

A theater is a large space, but floor area per person is only 0.5‐1 m^2^.^61^ If large audiences gather here, it will become a high‐density space. When audiences are calm, it is assumed that there will be no increase in the generation of droplets. However, since many and unspecified people occupy the same space for a long time, social distancing is important. Although it has the ventilation equipment set to the maximum audience numbers allowed, to avoid a high‐density situation, management of air change rate and the air filter is performed similar to that in the office scenario mentioned above. In the case of high density of people or audience behavior that will generate droplets, control ventilation, temperature, and RH become more important. Therefore, enough outdoor air needs to be introduced. Furthermore, if the interval time of movies or theaters showing is set, the indoor pollution concentration can be effectively reduced by ventilation.

### 3.5 Japanese style restaurant, karaoke room, club, live house, gym, etc

Japanese style restaurants, karaoke rooms, clubs, live houses, and gyms are high‐density people spaces. Since droplets increase due to accompanying activities, control of ventilation, temperature, and RH becomes more important. Although the HVAC systems of these institutions differ depending on the scales of a building, enough outdoor air needs to be introduced. Moreover, in a space with a small area, or a single room, use of an air purifier is effective as an auxiliary facility.

### 3.6 Medical facilities

In Japan, hospital infections (cluster infections) of COVID‐19 are currently occurring frequently. In foreign countries, SARS‐CoV‐2 was detected on various surfaces and indoor air in hospitals.^10^, ^11^, ^14^ It pointed that the measures by the side of environment, that is, HVAC systems, are important. The role of hospital HVAC systems is to mitigate the spread of airborne contaminants that could cause infections.^61^

In a hospital, there are rooms for special uses such as the operating room, the central supply room, etc. There is also the space which many people occupy, such as the visitors' waiting rooms, consultation rooms, and wards. Therefore, it is important that the design and management of the HVAC system of a hospital corresponds to the demands of cleanliness in the specific zones. A hospital facility design guideline has been published for the design and management of a hospital HVAC system.^62^ It provides recommendations for minimum total air change rates, circulation air rates, the main filter filtration efficiency, and pressure (positive, isobaric, negative) according to the classes of cleanliness required. Hospitals can be different from other buildings in that room pressure is set up, for protecting patients and staff from adverse currents of contaminants moving from a zone of low cleanliness class to a zone classified for high cleanliness. Therefore, when natural ventilation is carried out it is necessary to be aware of polluted air moving into a pure zone when the air balance breaks down. Similarly, dilution by ventilation, filtration with a filter, and an airflow plan (room differential pressure) are presented in the HVAC Design Manual for Hospitals and Clinics of ASHRAE (American Society of Heating, Refrigerating and Air‐Conditioning Engineers meeting) for controlling air cleanliness (Figure 4). Moreover, sterilization with ultraviolet rays is recommended in this manual as an auxiliary facility.^62^

Temperature and relative humidity control will differ for the different rooms. A temperature of 26°C and an RH of 50% are recommended by HEAJ for waiting rooms, wards, and the management department section.^63^

### 3.7 Elderly facilities

The facilities for the elderly are not defined as “specific buildings” by the Act on Maintenance of Sanitation in Buildings. Therefore, maintenance and management based on Standards for Environment and Health Management of Buildings is not required for the elderly facilities. However, special attention is required for the elderly as they become more severely affected when they develop COVID‐19.

The types of facilities for the elderly are diverse, and the scale and spatial composition of the facilities also vary. Recently, facilities for the elderly are oriented toward creating “homes.” Ventilation and air‐conditioning equipment is often similar to that of home; therefore ventilation and outside air intake should be based on measures taken at home. However, in common spaces where a large number of people often gather, such as a dining room and a day room, it is desirable to respond according to the measures mentioned for taverns, karaoke rooms, etc.

Regarding contact and droplet infections, it is inevitable that the inhabitants and staff will come into close contact with each other at the elderly facilities. Therefore, basic measures such as hand washing must be instituted.^64^

### 3.8 Houses

It is necessary to keep all the rooms well‐ventilated for infection control in houses. The installation of a 24‐hour ventilation systems to newly built houses has been obligatory since the Building Standards Law was enforced in July, 2003. In addition, in most houses in Japan, fans are installed in toilets and bathrooms. The operation of these systems and fans ensures some ventilation rates, but it is unclear if enough ventilation rates can be obtained through these systems and fans, so it is desirable to secure more ventilation by opening windows or doors.

[Houses equipped with 24‐h ventilation systems]

In houses with 24‐hour ventilation systems, the following is required.

- to keep the ventilation systems in operation
- to refer to the manual or contact the housing company if you do not know whether or not a 24‐hour ventilation system is installed to your house
- to keep an operation switch on
- to keep the ventilation power strong if there is a ventilation power switch
- to keep air intakes and outlets open and to clean the filters regularly

The ventilation rates of a 24‐hour ventilation system are designed as 0.5 times or more per hour, or 0.7 times or more per hour. This means that the all the air of a room is changed once in 2 hours; therefore, 24‐hour operation of the fans in toilets and bathrooms, and opening of windows are recommended for infection control even under when operating ventilation systems for 24 hours. If ventilation of a few dozens of times per hour is secured by opening windows, indoor air will be as fresh as outdoor air in a few minutes (If fresh air taken in is 3 times the volume of indoor air, 95% of indoor air will be changed).

[Houses without 24‐hour ventilation systems]

If a 24‐hour ventilation system is not installed to a house, fans in a toilet and a bathroom should be operated all the time and windows should be opened regularly.

If there is only one window in a room, it is also necessary to open the door of the room and the windows of other rooms to ensure air flow, and to improve the effectiveness of cooling fans or ventilation fans. In addition, it should be noted that general air‐conditioners for houses can circulate air but they do not work as ventilators.

### 3.9 Outdoor space, such as a park and an amusement park

There is no air‐conditioning/ ventilation equipment in an outdoor space. Even if SARS‐CoV‐2 is present, it is thought that the risk of infection is low, as it spreads and dilutes in large spaces. However, social distancing is important near a patient, since high concentration droplets may infect them before dilution occurs.

## 4 Ventilation and Cooling Based on Measures Against Heat Stroke

### 4.1 Room temperature, ventilation and cooling in summer

The following two items are important for the prevention of heat stroke: (i) proper use of air‐conditioners and fans to keep the room temperature below 28°C, and (ii) checking the room temperature frequently.^65^, ^66^ For the prevention of COVID‐19, it is recommended that where there is a window, it should be opened in two directions so that the wind can flow, and ventilation must be carried out at least twice every hour for several minutes.^67^ If the room temperature exceeds 28°C by opening the window and ventilating, the set temperature of the air‐conditioner must first be lowered and the situation must be monitored. If the room temperature is still high, it is important to keep the room temperature below 28°C by reducing the time during which the window is open, and the number of times the window is opened. Ventilation is important, but if there is a forecast for a hot summer day (a day with a maximum temperature of 30°C or higher), or a hot day (a day with a maximum temperature of 35°C or more), opening the window will result in the room temperature being the same as the high temperature outside, which is dangerous from the viewpoint of heat stroke. If the duration and number of window openings cannot be reduced, it is necessary to keep a distance of 2 m or more from others so that the indoor space is not crowded with many people near each other. In addition, it is necessary to take care in close‐contact settings such as close‐range conversations by wearing a mask when talking, and refraining from talking while commuting on public transport.

In buildings with mechanical ventilation and air‐conditioning equipment to secure the ventilation volume specified by law, such as air‐conditioning for the whole building and commercial air‐conditioners, temperatures should not exceed 28°C, which is the environmental hygiene control standard value set by law. In addition, it is important to maintain and manage mechanical ventilation equipment and air‐conditioning equipment, and to prevent COVID‐19 by opening windows and doors appropriately so that the temperature does not exceed 28°C.

### 4.2 Preventive measures against heat stroke indoors (The original versions are in the “Guideline for prevention of heat stroke in daily life”^65^ and “Prevention of heat stroke in daily life”^65^ of the Japanese Society of Biometeorology.)

#### 4.2.1 Measures for staying cool indoors

##### Block solar radiation heat entering the walls and windows facing west

In the summer, it is necessary to be careful, as walls and windows facing west are exposed to sunlight in the afternoon when the outside temperature rises. If the building has windows, the solar radiation heat to which the frame and windows of the building is exposed may be reduced by using blinds, reed screens, or a “green curtain” made of plants.

##### Use ventilation

Opening both a window in the main direction of the wind blowing in the area and a window in the opposite direction makes it easier for air to pass through. In addition, even if the wind is weak, if opposite windows of different heights, such as tall windows and those in stairwells are opened, the wind entering through the low windows passes through to the high windows, and the heat accumulated in the upper parts can be discharged.

##### Use air‐conditioners

There are studies showing that more than half of heat strokes occur at home (in the living room). Because heat often cannot be kept out without an air‐conditioner in modern cities, it is important to use an air‐conditioner without putting up with the heat. Since the actual room temperature and the set temperature of the air‐conditioner are different, it is important to check the room temperature with a thermometer. As a measure against COVID‐19 infection, if the window is open, the set temperature of the air‐conditioner may be lowered. However, be careful not to get too cold. When going out, it is advisable to check the temperature difference between inside and outside, so that the temperature difference does not cause a heat shock that thermophysiologically affects the human body. If the outdoor temperature is too high, one measure to prevent heat stroke may be to refrain from any nonessential and non‐urgent outings. In addition, the heat accumulated in the building during the day is gradually dissipated at night, so the incidence of heat strokes at night is also increasing. If it is hot and you cannot sleep, it is a good idea to cool the room before you go to bed, and allow it cool before you close the windows. Use the air‐conditioner when the outside temperature does not drop significantly at night. Before using a full‐scale air‐conditioner, make sure to have a trial run. Before using an air‐conditioner that has not been used for a long time, clean it by removing dust. It is also important to check whether the remote control is in proper operating condition, and whether cool air blows from the air‐conditioner.

##### Be careful even in high humidity

When selecting a thermometer to check the room temperature, it is recommended to select one with a humidity display. When the humidity is high, it is easy for the body to retain heat. A little lower indoor temperature makes it easier to spend time indoors. Also, even if you do not think you are sweating, water is always lost from the surface of your body. It is important to rehydrate before you feel thirsty, even if you are in a room with air‐conditioning.

##### Promote heat dissipation from the body through clothing

Outdoors, you can prevent heat by using a parasol or wide‐brimmed hat to control the exposure of your skin and block sunlight. Indoors, it is easier to dissipate heat from the body by choosing clothes with large skin exposure (small clothing area), such as a combination of a tank top and shorts. Also, the air inside the clothes is released from the neck and sleeves of the clothes. In particular, air is easily released from clothes having large openings vertically above and below. Not only the shape of the clothes but also the nature of the material is important, and in order to release high temperature and high humidity air inside the clothes, it is also effective to put on clothes made of a material with good breathability to release heat. Indoors, you should select clothes that are made of a material that easily dissipates heat from your body, and easily evaporates sweat. However, you need to be careful if your body is too cold.

#### 4.2.2 Matters requiring special attention

##### People who are easily affected by heat stroke

There are studies showing that the elderly, infants, school children, those who work too hard at their job or sports, those who wear heavy clothing at work, those who have a chronic illness, those who are obese, and those who are unwell, are more likely to develop heat stroke. More aggressive measures are needed to prevent heat stroke in those people. There are studies showing that bedridden people also easily develop heat stroke. Also, when there are people who have underdeveloped or low thermoregulatory functions, it is necessary for people around to understand their weakness with respect to heat, and be careful. In some cases, visits and telephone confirmations may be necessary.

### 4.3 Indoor heat stroke prevention measures based on COVID‐19 precautions

The following measures based on COVID‐19 precautions will be added to the heat stroke prevention leaflet proposed by the Ministry of Health, Labor and Welfare.^68^

#### 4.3.1 Avoid getting hot

1. Use the air‐conditioner and the fan well so that the room temperature is below 28°C
  - In the case of room with windows
    - If the room temperature exceeds 28°C, opening the window and ventilating must be done first, but before that lower the set temperature of the air‐conditioner.
    - If the room temperature still exceeds 28°C, set the temperature of the air‐conditioner lower. You should reduce the time during which the window is open, and the number of times the window is opened.
    - If there is a forecast that it will be a hot summer day (a day with a maximum temperature of 30°C or higher), or a hot day (a day with a maximum temperature of 35°C or more), you should reduce the time during which the window is open, and the number of times the window is opened, and use the air‐conditioner and the fan, to prevent the room temperature from reaching the same level as the outside temperature.
    - If there is a forecast that it will be a tropical night, you should cool the room with an air‐conditioner before going to bed.
    - If you ventilate by opening windows, you should open both windows to allow wind in and out. (If you open both a window in the main direction of the wind blowing in the area and a window in the opposite direction, it can be easier for air to pass through. Also, if you open opposite windows of different heights, such as stairwells and tall windows, the heat accumulated in the upper part can be discharged.)
    - If the duration and number of window openings cannot be reduced, it is necessary to keep a distance of 2 m or more from others so that the indoor space is not crowded with many people near each other.
    - In addition, it is necessary to take care in close‐contact settings such as close‐range conversations by wearing a mask when talking, and refraining from talking while commuting on public transport.
  - In the case of a room with mechanical ventilation and air‐conditioning equipment
    - In buildings with mechanical ventilation and air‐conditioning equipment to secure the ventilation rates specified by law, such as air‐conditioning for the whole building and commercial airconditioners, do not exceed 28°C, which is the environmental hygiene control standard value set by law. In addition, it is important to maintain and manage mechanical ventilation equipment and air‐conditioning equipment, and to prevent COVID‐19 by opening windows and doors appropriately so that the temperature does not exceed 28°C.
    - Before using a full‐scale air‐conditioner, make sure to do a trial run.
2. Check the room temperature frequently.
  - If you feel the heat reducing, you should check the room temperature with a thermometer and not rely only on experience.
  - When selecting a thermometer to check the room temperature, it is recommended to select one with a humidity display. You should use the air‐conditioner and the fan, if the humidity is high, as it is easy for the body to retain heat when it is humid.
  - When going out, it is advisable to check the temperature difference between the inside and outside, so that the temperature difference does not cause a heat shock that thermophysiologically affects the human body. If the outdoor temperature is too high, one measure to prevent heat stroke may be to refrain from any nonessential and non‐urgent outings.

#### 4.3.2 Frequently rehydrate

Even if you feel that you are not sweating, water is always lost from the surface of your body. Therefore, it is important to rehydrate even before you feel thirsty, and even if you are in a room with the air‐conditioner.

#### 4.3.3 Important points

- Please do not just think that “it's okay,” as the way you feel the heat changes depends on your physical condition on each particular day.
- Because there are studies showing that the elderly, infants, school children, those who work too hard at their job or sports, those who wear heavy clothing at work, those who have a chronic illness, those who are obese, and those who are unwell, are more likely to develop heat stroke, more aggressive measures are needed to prevent heat stroke in those people.
- When there are people who are bedridden or have underdeveloped or low thermoregulatory functions, it is necessary for people around to understand their weaknesses with respect to heat, and to be vigilant. In some cases, visits and telephone confirmations may be necessary.

## 5 Conclusion

**Figure 5:**
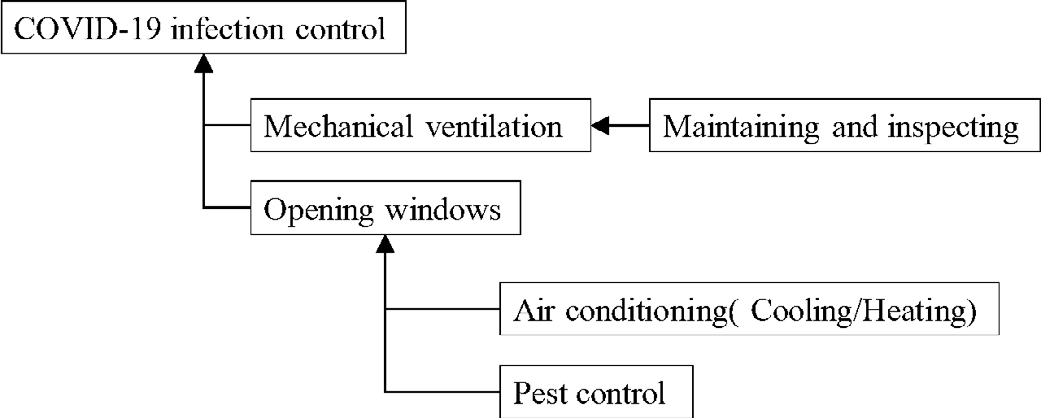
Ventilation measures for summer air‐conditioning

This report summarizes ventilation measures for summer air‐conditioning based on the latest evidence collected concerning COVID‐19 as shown in Figure 5. Some recommendations concerning air‐conditioning and ventilation are shown for various situations because it is difficult to specify standard values of ventilation rates etc. from the evidence presently available. Furthermore, the following measures are recommended.

[In every indoor space]

- Enough ventilation is necessary to prevent the infection of COVID‐19.
- Opening windows is an effective ventilation method and it is desirable to open them wider and for a longer time.
- In summer, air‐conditioning is essential for good health, for example, for heat stroke prevention. (In winter heating is essential for good health, for example, for heat shock.)
- General air‐conditioners do not function as ventilators, so mechanical ventilation or opening windows is necessary.
- When windows are open, it is necessary to prevent animals or insects pests from coming in.

[In case an air‐conditioner and a ventilation system are equipped]

- It is necessary to ensure the operation of designed ventilation rates by maintaining and inspecting the equipment.
- It is recommended to control the number of people in a room, to assure the ventilation rate for a person and to shorten people's stay in a room.
- Measures as the improvement of ventilation effects through the development of air‐conditioners and ventilation systems, and the use of air cleaners and humidifiers in winter have to be discussed considering the characteristics of each building, that is, what it is used for, how often it is used, or what kind of air‐conditioner or ventilation system it has.

## Disclosure

The authors have no conflict of interest.
